# Harnessing the natural anti-glycan immune response to limit the transmission of enveloped viruses such as SARS-CoV-2

**DOI:** 10.1371/journal.ppat.1008556

**Published:** 2020-05-21

**Authors:** Adrien Breiman, Nathalie Ruvën-Clouet, Jacques Le Pendu

**Affiliations:** 1 Université de Nantes, Inserm, CRCINA, Nantes, France; 2 CHU de Nantes, Nantes, France; 3 Oniris, Ecole Nationale Vétérinaire, Agroalimentaire et de l’Alimentation, Nantes, France; University of Pittsburgh, UNITED STATES

Enveloped viruses are glycosylated, meaning that their envelope proteins are post-translationally modified by the addition of glycans. Some glycosyltransferases that contribute to terminate glycan chains synthesis are differentially expressed between cell types within or between species, which bears important immunological consequences [1]. A prime example concerns the *GGTA1* gene encoding a galactosyltransferase that catalyzes the transfer of a galactose in α1,3 linkage onto subterminal N-acetyllactosamines. This glycosyltransferase is expressed by all mammals except Old World monkeys, apes and humans, due to inactivating mutations that occurred in a common primate ancestor some 20 to 40 million years ago. Consequently, all humans lack the αGal glycan motif and possess natural anti-αGal antibodies generated in response to bacteria of the microbiota that express similar glycans [2]. Likewise, humans lack expression of the N-glycolyl form of sialic acid (NeuGc) due to a pseudogenization event of the *CMAH* gene that occurred about 2 million years ago. In most other animal species, the orthologous gene encodes the cytidine monophosphate (CMP)-NeuAc hydroxylase that converts NeuAc into NeuGc from the nucleotide form CMP-NeuAc. As a result of our inability to synthesize NeuGc, natural anti-NeuGc are also present in humans (reviewed in [3,4]). Another example concerns the enzymes that are involved in the synthesis of the ABO histo-blood group antigens. The A and B enzymes catalyze the transfer of an N-acetylgalactosamine and a galactose, respectively, in α1,3 linkage on a precursor structure called the H antigen, generating the corresponding A or B antigens. They are encoded by distinct alleles at the *ABO* locus. The O alleles are null alleles responsible for a lack of transferase, in which case the H antigen remains unchanged. O alleles in the homozygote state confer blood group O, which is characterized by a complete absence of A or B antigens [5]. Under stimulation by bacteria of the microbiota that present glycan motifs similar to either A or B antigens, blood group O people develop so-called “natural” anti-A and anti-B antibodies, whilst blood group A and B individuals develop either anti-B or anti-A antibodies, respectively [6]. Only people of the AB subgroup lack such antibodies. In humans, besides their expression on red blood cells, ABH antigens are widely expressed on many other cell types, including vascular endothelial cells and epithelial cells of many organs [7]. Importantly, the titers of anti-αGal, anti-NeuGc, and anti-A/B antibodies are highly variable between individuals, ranging from 100- to 1000-fold [8,9].

When enveloped viruses are produced by cells expressing these glycan epitopes, they can be effectively neutralized by anti-αGal or anti-A and anti-B antibodies as shown for several animal and human enveloped viruses (reviewed in[2]). Coronavirus S protein trimers are covered by an extensive glycan shield made of N-linked glycans that surrounds the receptor-binding domain [10]. The recently emerged SARS-CoV-2 responsible for COVID-19 shows overall conservation of the S protein glycosylation sites. The primary target organ of human coronaviruses, including both SARS and SARS-CoV-2, is the lung and both viruses use angiotensin converting enzyme 2 (ACE2) as receptor [11]. Being expressed on lung alveolar epithelial cells, chiefly type 2 pneumocytes, [12,13], it is to be expected that the glycosylation of SARS-CoV and SARS-CoV-2 should be similar.

Using a cellular experimental model, our group showed that the interaction between SARS-CoV S protein and ACE2 could be specifically blocked in a dose-dependent manner by anti-A blood group antibodies when the S protein was synthesized by cells that expressed the A histo-blood group antigen following transfection by the appropriate glycosyltransferases cDNA [14]. These observations suggested that, when produced in cells that express the A or B blood group enzymes, infectious SARS virions are decorated by the corresponding glycan antigens and that the presence of anti-A and anti-B antibodies in blood group O individuals could prevent infection by blocking virus attachment and entry.

**Fig 1:**
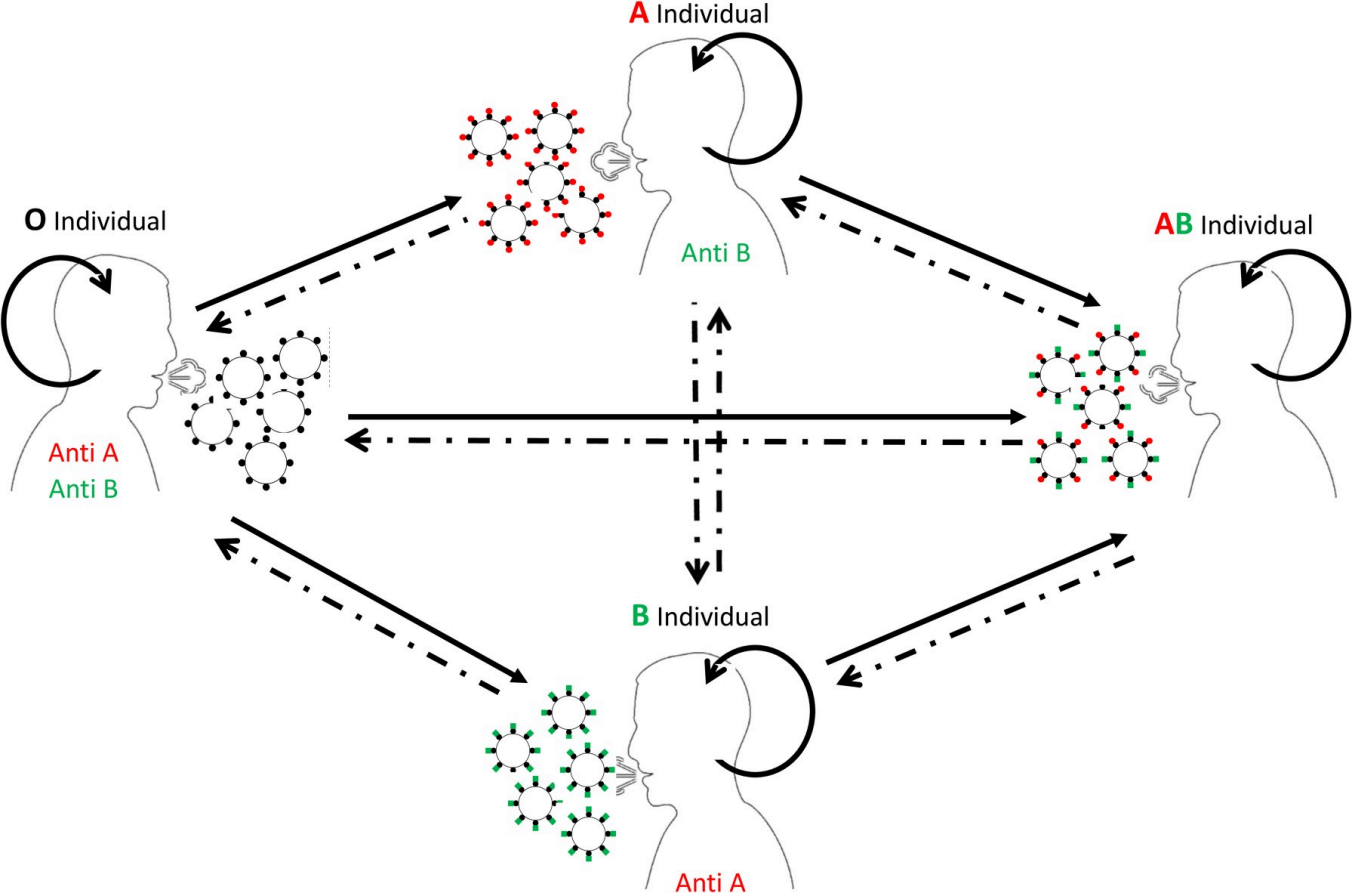
Virus transmission pattern in the presence of an ABO blood group effect. Virions produced by blood group O individuals are devoid of A or B antigens and can be fully transmitted regardless of the recipient blood type (full arrows). Viruses produced by A and B blood groups individuals are decorated by A or B blood group epitopes (red and green spikes, respectively) and viruses produced by blood group AB individuals are decorated by both A and B epitopes. Transmission of such viruses will be decreased by the presence of either anti-A and/or anti-B of the recipient (dashed arrows). Transmission between individuals of the same subtype will always be maximal (circular arrows). In the presence of high-titered anti-A and anti-B antibodies, transmissions represented by dashed arrows should be completely ablated.

Moreover, blood group O individuals were at a much lower risk of being infected than non-O individuals in a Hong Kong 2003 SARS hospital outbreak [15], and a similar trend has just been observed for COVID-19 in China [16]. Accordingly, blood group O individuals would be at a lesser risk of being infected than non-O individuals due to blocking of potential transmission events from either A, B, or AB individuals, providing anti-A or anti-B titers are of sufficient magnitude (Fig 1). Mathematical modeling of the consequences of this potential limitation of virus transmission suggested that the Hong Kong SARS hospital outbreak had been slowed down to some extent thanks to the ABO genetic polymorphism and the ensuing neutralizing anti-A and anti-B antibodies. It further indicated that if anti–blood group A and/or B titers had always been high, transmission of the virus, in the absence of any containment measure, would be largely impaired and the outbreak slowed to a considerable extent [14].

We therefore hypothesize that as they are produced in cells coexpressing the ACE2 receptor and either the αGal, NeuGc, or A/B blood group antigens, both SARS-CoV and SARS-CoV2 harbor the corresponding glycan epitopes. Because of the natural immune response against these epitopes, the αGal and NeuGc xenoantigens would contribute to prevent cross-species transmission from nonprimate mammals to humans, while A/B blood group antigens would contribute to decrease and slow between-human transmission. Nonetheless, owing to the presence of individuals with low anti-αGal titers, occasional cross-species transmission may occur. Interestingly, a recent genomic analysis across vertebrates revealed that two bats lineages, including *Rhinopholus* bats suspected to have originated the SARS-CoV-2 closest ancestor, lost their *Cmah* gene function, similar to humans [17]. The lack of NeuGc xenoantigen on the virions produced by these bats might have facilitated cross-species transmission. Likewise, impairment of transmission by the anti–blood group antibodies may not work to its full potential because of their variable titers in the population and of the high affinity of the SARS-CoV2 for ACE2 [18], rendering its neutralization more difficult. This leaves room to amplify these innate mechanisms of protection in preparation for the next emergence and mitigation of the virus impact once emergence has occurred.

If the antibody blocking effect can be documented in vitro, and possibly in vivo, it will become important to consider raising the anti-αGal, as well as the anti-A and anti-B antibodies titers in human populations. That could be achieved as previously described either by immunizing against inactivated harmless bacteria that harbor the αGal, A, and B epitopes or by immunizing against the corresponding synthetic oligosaccharides linked to an immunogenic scaffold [19,20]. Raising the anti-A and anti-B titers in the whole population carries the risk of complicating incompatible platelet transfusion as well as increasing the risk of hemolytic disease of the newborn in case of mother–infant ABO incompatibility. These issues should be carefully dealt with. Raising the anti-NeuGc titers might be more problematic since meat and dairy products consumption allows incorporation of NeuGc onto human glycans, and this may contribute to the promotion of inflammation and cancer progression as experimentally demonstrated [3,21]. By contrast, raising the anti-αGal titers should not carry any risk since the antigen is entirely absent from human tissues.

Blood groups A and B might also be harnessed to increase the efficacy of SARS-CoV-2 vaccines. Indeed, the virus spike proteins, which are the main target of currently designed vaccines, might be produced in cells that are enzymatically equipped to synthetize A and B antigens so that the vaccine glycoprotein will carry these epitopes. In addition to generating neutralizing anti-S protein, the vaccine would stimulate anti-A and anti-B responses that may contribute to the vaccine efficacy in all cases of ABO incompatible transmissions.

In conclusion, we propose to enhance the innate anti-viral protection conferred by natural anti-glycan antibodies in order to lower both the risk of emergence of coronaviruses, or other enveloped viruses, from a nonprimate mammalian species and the risk of transmission within the human population. This could add-up to other protection and containment measures, mitigating the impact of the epidemic.
